# Assessing the Effectiveness of Interventions Implemented by Nurses to Reduce Medication Administration Errors in Hospitalised Acute Adult Patient Settings: Systematic Review and Meta‐Analysis

**DOI:** 10.1111/jocn.70109

**Published:** 2025-10-01

**Authors:** Angela Uche Eze, Takawira Marufu, Albert Amagyei, David Nelson, Despina Laparidou, Joseph C Manning

**Affiliations:** ^1^ Nottingham University Hospitals NHS Trust University of Lincoln Lincoln UK; ^2^ Nottingham Children’s Hospital Nottingham University Hospitals NHS Trust Nottingham UK; ^3^ Nottingham University Hospitals NHS Trust Nottingham UK; ^4^ Lincoln International Institute for Rural Health (LIIRH). Lincoln UK; ^5^ Community and Health Research Unit (CaHRU) University of Lincoln Lincoln UK; ^6^ School of Healthcare, College of Life Sciences University of Leicester Leicester UK

**Keywords:** adult inpatient, effectiveness, hospitals, medication administration errors, meta‐analysis, nurses, systematic review

## Abstract

**Background:**

Medication administration errors are high‐risk patient safety issues that could potentially cause harm to patients, thereby delaying recovery and increasing length of hospital stay with additional healthcare costs. Nurses are pivotal to the medication administration process and are considered to be in the position to recognize and prevent these errors. However, the effectiveness of interventions implemented by nurses to reduce medication administration errors in acute hospital settings is less reported.

**Aim:**

To identify and quantify the effectiveness of interventions by nurses in reducing medication administration errors in adults' inpatient acute hospital.

**Methods:**

A systematic review and meta‐analysis was conducted up to 03/24. Six databases were searched. Study methodology quality assessment was conducted using the Joanna Briggs Institute (JBI) critical appraisal tools, and data extraction was conducted. Meta‐analysis was performed to combine effect sizes from the studies, and synthesis without meta‐analysis was adopted for studies that were not included in the meta‐analysis to aggregate and re‐examine results from studies.

**Results:**

Searches identified 878 articles with 26 studies meeting the inclusion criteria. Five types of interventions were identified: (1) educational program, (2) workflow smart technologies, (3) protocolised improvement strategy, (4) low resource ward‐based interventions, and (5) electronic medication management. The overall results from 14 studies included in meta‐analysis showed interventions implemented by nurses are effective in reducing medication administration errors (*Z* = 2.15 (*p* = 0.03); odds ratio = 95% CI 0.70 [0.51, 0.97], *I* ^2^ = 94%). Sub‐group analysis showed workflow smart technologies to be the most effective intervention compared to usual care. Findings demonstrate that nurse‐led interventions can significantly reduce medication administration errors compared to usual care. The effectiveness of individual interventions varied, suggesting a bundle approach may be more beneficial. This provides valuable insights for clinical practice, emphasizing the importance of tailored, evidence‐based approaches to improving medication safety.

**Reporting Method:**

PRISMA guided the review and JBI critical appraisal tools were used for quality appraisal of included studies.

**Summary**

- What does this paper contribute to the wider global clinical community?
  ○ This systematic review and meta‐analysis provide an update on evidence‐based insights into nurse‐led interventions that reduce medication administration errors in hospitalised acute adult patients.
  ○ It will contribute to global clinical practice by standardising best practices, informing nursing education, enhancing patient safety, and guiding healthcare policies.
  ○ The study demonstrates the importance of innovation and investment in technology, highlights the role of nurses in medication safety, and offers globally relevant recommendations.
  ○ It also identifies research gaps, encouraging further studies and quality improvement initiatives to enhance medication safety worldwide.

## 1 Introduction

Unsafe medication practices and medication errors are a leading cause of injury and avoidable harm in health care systems across the world (World Health Organization 2017a; Tsegaye et al. 2020). Medication errors are described as any Patient Safety Incidents (PSI) where there has been an error in the process of prescribing, preparing, dispensing, administering, and monitoring or providing advice on medicines (NHS England 2014). Medication errors can occur at any point along the care pathway (NHS England 2022), however, recent studies indicate that medication administration errors (MAE) persist as a significant category of medication errors in clinical settings (Samsiah et al. 2020; Kerari and Innab 2021; Mulac, Mathiesen, et al. 2021), and nurses are often responsible for medication administration to patients (Schroers et al. 2021; Hanson and Haddad 2023).

Medication administration error is defined as a deviation from the prescriber's medication order as written on the patient's chart, manufacturers' preparation/administration instructions, or relevant institutional policies (Keers et al. 2013). There are various unwanted consequences of medication errors for both patients and health professionals. This includes harm to the patient, delaying recovery, increased length of hospital stay with additional healthcare costs, and poor quality of life (Elliott et al. 2018). Increasingly complex medical needs and the introduction of many new medications have resulted in Adverse Drug Events being recognized as a key global issue (Elliott et al. 2021).

These incidents led to the launching of World Health Organisation's third global patient safety challenge “Medication without Harm” which aimed to reduce the global level of severe, avoidable harm related to medications by 50% between 2017 and 2022 (World Health Organization 2017b). The challenge laid out strategies to improve the way medicines were prescribed, distributed, and consumed, including awareness among patients about the risks associated with the improper use of medication (World Health Organization 2017a; Care Quality Commission 2019). This initiative was adopted in the UK through the National Patient Safety Strategy together with the Medicines Safety Improvement Programme to address/reduce the most important causes of severe harm associated with medicines by 50% by March 2024 (NHS England 2022). Nurses are not only the largest group of health care professionals who administer medications; however, they are considered to be in the best position to recognise and prevent medication errors before patient safety is compromised (Flynn et al. 2012; European Biosafety Network 2024).

The aim of this systematic review and meta‐analyses is to identify and quantify the effectiveness of interventions by nurses in reducing medication administration errors in adults' inpatient care in acute hospitals. This will contribute to the ongoing effort to enhance medication safety, minimise medication administration errors, and improve overall patient care in acute hospital settings.

## 2 Methods

### 2.1 Design

This systematic review was conducted in line with the Joanna Briggs Institute (JBI) methodology for systematic reviews of effectiveness (Tufanaru et al. 2017) and in adherence with the Preferred Reporting Items for Systematic Reviews and Meta‐Analyses guideline (https://www.prisma‐statement.org/) to ensure quality and completeness (Supplementary file—PRISMA_2022_Checklist). The review protocol was registered with the International Prospective Register of Systematic Reviews (PROSPERO) prior to conducting active searches (available at https://www.crd.york.ac.uk/prospero/display_record.php?ID=CRD42024519302).

### 2.2 Search Methods

The searches were conducted across six databases using a pre‐determined search strategy (Appendices S1 and S2): MEDLINE, EMBASE, CINAHL, Web of Science, The Cochrane Library, and ClinicalTrials.gov, using a combination of various keywords and Medical Subject Headings (MeSH terms). Search terms included ‘adult’, ‘acute’, ‘hospital’, ‘inpatient’, ‘nurse’, ‘medication administration’, ‘drug’, ‘medicine’, ‘drug’, ‘prevention’, ‘intervention’, ‘strategies’, ‘multidisciplinary approach’, ‘errors’, ‘incident’, ‘adverse drug events’, ‘harm’, ‘datix’, ‘risk’, ‘safety’, ‘reduction’, and ‘error rate’. The search was constructed using the Population Intervention Comparator and Outcome (PICO) model by combining the search terms using Boolean operators ‘OR’ and ‘AND’ where necessary. There were no restrictions on the publication timeframe for selected studies. Due to the lack of translation services, only studies published in the English language were included.

### 2.3 Inclusion and/or Exclusion Criteria

This review considered all primary quantitative research studies: Randomised Control Trials (RCTs), experimental studies, quasi‐experimental studies, pre and post‐test studies for the assessment of harms, interrupted time series analysis, evaluation studies, and studies that measured nurses' MAE rates before and after the implementation of the intervention.

The context involved acute hospital settings in which adult (18 years and above) patients are admitted and receive medication administered by nurses. Studies were also included if they involved testing of an intervention implemented by nurses aimed at reducing MAEs, and the rate of MAEs must be identified as a primary or secondary outcome to be included.

Studies were excluded if they were conducted in care homes, community‐based geriatric settings, institutional settings caring for elderly persons, psychiatric institutions, and inpatient settings that provided care to children. In addition, studies were excluded if the rate of MAEs was not specified in the outcomes.

### 2.4 Study Selection

Following the search, all identified citations were collated and uploaded to Covidence, the software for managing systematic reviews (Harrison et al. 2020). After duplicate removal, two reviewers (AE and AA) independently screened titles and abstracts against the inclusion and exclusion criteria. The studies that met the inclusion criteria were retrieved in full and imported into Covidence. Full‐text articles that did not meet the inclusion criteria were excluded with reasons provided.

### 2.5 Quality Appraisal

Methodology quality assessment was done independently by two authors (AE and AA) using the JBI Critical Appraisal Tool for Cross Sectional Studies, Cohort Studies, Case Control Studies, Quasi‐Experimental studies, and Randomised Control Trials (Munn et al. 2020). Studies were not excluded based on the low score obtained. Disagreements between the two authors were resolved by including a third reviewer.

### 2.6 Data Extraction

Data were extracted by the first reviewer (AE) using a standard form that included the author's name, year of publication, country of study, study design, setting, sample size, intervention type, effects, and outcomes obtained for the intervention strategies. The second reviewer (AA) then checked the data for accuracy; discrepancies were discussed, and corrections were made appropriately. In addition, corresponding themes and findings related to the phenomena of interest were extracted. The data extraction form was adapted from JBI Critical Appraisal Checklist for Systematic Reviews and Research Syntheses (Joanna Briggs Institute 2017).

### 2.7 Data Synthesis

The primary outcomes included the rate of errors (administration error rates) and the effect of interventions implemented by nurses on administration error rates. These were measured as the number of errors relative to the total opportunity for error. Additional outcomes were types of MAEs and their clinical impact. Secondary outcomes included, where possible, the severity of MAEs that cause harm to patients such as adverse drug events, mortality, morbidity, length of hospital stay, and quality of life, as well as barriers and facilitators of the implementation of improvement strategies.

The first reviewer summarised the characteristics of the included reviews in tables and presented estimates of the reduction in medication error rate for each intervention. A meta‐analysis was performed to systematically combine and compare all available data on the effectiveness of interventions implemented by nurses in reducing MAEs in hospitalised acute adult patient settings from the quantitative empirical studies so as to estimate the average summary effect of the results (Cheung and Vijayakumar 2016). Studies with statistical data presented as OR and/or provided relevant data for calculation of OR were included in the meta‐analysis. Fourteen studies involving 2034 participants met these criteria and were included in the meta‐analysis. A synthesis without meta‐analysis (narrative synthesis) was adopted for studies that were not included in the meta‐analysis to aggregate findings of the multiple studies (Willig and Rogers 2017).

## 3 Results

**FIGURE 1:**
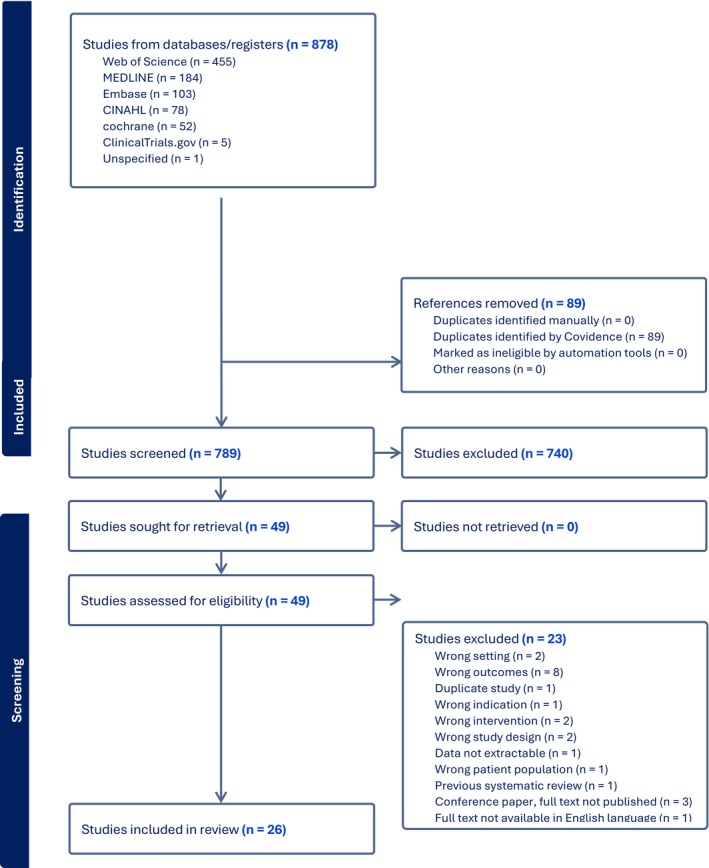
PRISMA Flow Diagram. [Colour figure can be viewed at wileyonlinelibrary.com]

The initial online search for studies identified 878 articles; following the removal of duplicates, 789 studies were screened at the title and abstract stages, and 740 citations were identified as irrelevant to the research topic. Forty‐nine (49) papers were assessed at full text, and 23 studies were excluded, as they did not meet the inclusion criteria (Figure 1 Prisma Flow Diagram shows a stepwise guide from identification to inclusion). Fourteen (14) studies were included for meta‐analysis, and 12 studies were reported using synthesis without meta‐analysis (narrative synthesis) (see Table 1 Characteristic of included studies).

**TABLE 1:** Characteristics of included studies.

Author and Year	Country	Type of study	Participants	Sample size (N)	Interventions	Results	Study Quality
Bergqvist et al. 2009	Sweden	Case control study	Adult patients and nurses (Division of Internal Medicine)	460—patients, 32—Nurses	Educational program; 1‐day training in clinical pharmacology	Of 460 patients (250 intervention group and 210 in the control group), 38% and 36%, respectively, had at least one re‐admission to hospital (*p* = 0.86), and 24% of the patients died. Eighteen% and 17% (43/37), respectively, used one or more inappropriate drugs (*p* = 0.90). The nurses found 86 clinically significant Drug Related Problems not detected by the usual care. There was no statistical difference in the number of drug‐related re‐admissions between the groups, 14/16, respectively (*p* = 0.40). (OR 1.26, 95% CI [0.81, 1.97])^a^	High
Booth et al. 2024	USA	Retrospective cohort study	Adult patients (community teaching hospital's suburban emergency department)	356—patients	Work‐flow Smart Technologies; The use of the unfractionated heparin infusion calculator and independent double check for UFH before administration	There were 13.9% errors (39 of 279) present when the calculator was used and 23.3% (18 of 77) when the calculator was not used (*p* = 0.046). There was 86% correct administration of heparin (240 of 279) when the calculator was used and 76% correct administration (59 of 77) when the calculator was not used^a^ Subgroup analysis on the types of MAEs observed was performed. The bolus dose administered was incorrect in 3.23% of cases (9 of 279) where the UFH calculator was used versus 3.85% of cases (3 of 77) where the UFH calculator was not used. The initial drip rates were incorrect in 3.94% of cases (11 of 279) where the calculator was used versus 10.26% of cases (8 of 77) where the calculator was not used. No baseline aPTT was collected in 7.17% of cases (20 of 279) where the calculator was used versus 8.97% of cases (7 of 77) where the calculator was not used. (OR 0.53, 95% CI [0.28, 1.00])^a^	High
van der Veen et al. 2020	Netherlands	Prospective observational study	Adult patients and nurses (4 general hospitals)	1230—patients 272—nurses	Work‐flow Smart Technologies; workarounds performed by nurses in barcode‐assisted medication administration	5793 medication administrations among 1230 patients given by 272 nurses. In 3633 (62.7%) of the administrations, one or more workarounds were observed. In the multivariate analysis, factors significantly associated with workarounds were the medication round at 02 p.m.–06 p.m. (adjusted odds ratio [OR]: 1.60, 95% CI: 1.05–2.45) and 06 p.m.–10 p.m. (adjusted OR: 3.60, 95% CI: 2.11–6.14) versus the morning shift 06 a.m.–10 a.m., the workdays Monday (adjusted OR: 2.59, 95% CI: 1.51–4.44), Wednesday (adjusted OR: 1.92, 95% CI: 1.20–3.07) and Saturday (adjusted OR: 2.24, 95% CI: 1.31–3.84) versus Sunday, the route of medication, non‐oral (adjusted OR: 1.28, 95% CI: 1.05–1.57) versus the oral route of drug administration	Low
Johnson et al. 2017	Australia	Cluster randomised controlled trial	8 wards from 2 metropolitan hospitals in Sydney (adult medical, surgical units including aged care, medical assessment, gastrointestinal, cardiology, rehabilitation, and orthopaedics)	534—patients	Low Resource Ward‐based Interventions; (1) nurse initiated‐recall cards; and (2) cross‐checking medication charts during nursing handover	There were no statistically significant differences in either of the omission rates between the control and intervention arms (omission rate without documentation: *p* = 0.25; omission rate of all omissions: *p* = 0.40) Further analysis using generalised estimating equations adjusting for hospital and patient age identified an effect for the intervention wards (Wald *χ* ^2^ = 9.439, df = 1, *p* = 0.002)	Moderate
Nash et al. 2005	USA	Prospective intervention study	Adult patients (3 units from Mount Sinai Hospital)	7608 doses administered to patients	Low Resource Ward‐based Interventions; Medication safety reporting system (MSRS)	The baseline rate of excessive dosing was 23.2% of administered medications requiring adjustment for renal insufficiency given to patients with renal impairment on the participating units and 23.6% in the rest of the hospital. The rate fell to 17.3% with nurse feedback and 16.8% with pharmacist feedback in the participating units (*p* < 0.05 for each, relative to baseline). The rates of excessive dosing for the same periods were 26.1% and 24.8% in the rest of the hospital. There was a significant reduction in the rate of excessive dosing in patients with renal insufficiency on the participating units with the nurse and pharmacist. (0.59, 95% CI [0.54, 0.65])^a^	Moderate
Westbrook et al. 2020	Australia	Controlled before and after study	2 adult teaching hospitals (3 intervention and 3 control wards)	7451 administrations were observed (4176 pre‐EMS and 3275 post‐EMS)	Electronic Medication Management; Implementation of electronic medication system	At baseline, 30.2% of administrations contained ≥ 1 MAE, with wrong intravenous rate, timing, volume, and dose being the most frequent. Post EMS, MAEs decreased on intervention wards relative to control wards by 4.2 errors per 100 administrations (95% CI 0.2 to 8.3; *p* = 0.04). Wrong timing errors alone decreased by 3.4 per 100 administrations (95% CI 0.01 to 6.7; *p* < 0.05); The size of reduction in serious MAEs attributable to the EMS was an absolute reduction in serious MAEs of 2.4% (95% CI 0.8% to 3.9%; *p* = 0.003). This equated to a 56% overall relative reduction in serious MAEs on the intervention wards. (OR 1.03, 95% CI [0.88, 1.21])^a^	High
Schimmel et al. 2011	Netherland	Prospective, observational study with a before–after design	30—bed orthopaedic ward (Erasmus university medical centre)	86—patients	Low Resource Ward‐based Interventions; Implementation of the second medication cart filling method by arranging medicines by their names	After the intervention, 170 of 740 (23.0%) medication administrations with one or more medication administration error(s) were observed compared to 114 of 589 (19.4%) before the intervention (odds ratio 1.24 [95% confidence interval 0.95–1.62]). The distribution of subtypes of medication administration errors before and after the intervention was statistically significantly different (*p* < 0.001) (OR 1.21, 95% CI [0.93, 1.57])^a^	Moderate
Redley and Botti 2013	Australia	Retrospective/descriptive analysis	2 hospital sites of a large non‐profit, private health service	359 incident reports	Electronic Medication Management; Effects of introducing an electronic medication management system	Of the 359 medication errors, most occurred at the nurse administration (71.5%) and prescription (16.4%) stages, however, prescription errors were more common at Site B (*n* = 41, 31.1%), and nurse administration errors were more common at Site A (*n* = 178, 78.4%). The most common medication error type reported at Site A was omission (33%), and at Site B was wrong documentation (24.2%). Most of the reported medication errors were associated with a mild impact on patient outcomes (Site A, *n* = 209, 92% and Site B, *n* = 117, 88.6%) and few had moderate (Site A, *n* = 16, 7% and Site B, *n* = 14, 10.6%) or severe (Site A, *n* = 2, 0.9% and Site B, *n* = 1, 0.8%) outcomes (OR 0.41, 95% CI [0.26, 0.66])^a^	High
Verweij et al. 2014	Netherland	Mixed methods before‐after study	3 adult wards in a Dutch 1024‐bed university hospital (neurology, neurosurgery, and a combined ward with dermatology, ophthalmology, and ENT service)	313 medication administrations	Low Resource Ward‐based Interventions; Implementation of drug round tabards	Significant reductions in both interruptions and MAEs were found after the implementation of the tabards. In the third period, a decrease of 75% in interruptions and 66% in MAEs was found. Linear regression analysis revealed a model R2 of 10.4%. The implementation topics that emerged can be classified into three themes: personal considerations, patient perceptions, and considerations regarding tabard effectiveness. Parameter Estimate for Intercept MAEs (*β* = 0.800, standard error = 0.065, Test statistics = 12.361, *p <* 0.05) and Variable Interruptions MAEs (*β* = 0.271, standard error = 0.045, Test statistics = 6.005, *p <* 0.05) (OR 0.00, 95% CI [0.00, 0.01])^a^	Moderate
Allison Rout et al. 2023	South Africa	Cross‐sectional study	3 adult private and public Intensive Care Units	223—Carbapenem infusion administrations to 20—patients	Low Resource Ward‐based Interventions; Observation of double‐checking process and infusion labeling practices	Nurses were observed carrying the additive medication label away from the patient's bedside to other nurses, requesting a second signature without checking the drug (vial), medication chart, and confirming the patient's identity before signing the label. Adherence to the scheduled time occurred in 34.9% of administrations, 5.4% of doses were not given, and an incorrect dose was given in 1.4% of administrations. One hundred and forty‐four (64.6%) infusion bags were inspected during the administrations: there was no medication label affixed to 21.5% of bags, and only 8.3% of bags were labelled with essential details	Moderate
Skog et al. 2022	USA	Observational Before and After Study	3 hospitals (community healthcare system)	350 infusions pre intervention (178 patients) and 367 post intervention (200 patients).	Work‐flow Smart Technologies; Implementation of smart pump interoperability	Total errors significantly decreased from 401 (114.6 per 100 infusions) to 354 (96.5 per 100 infusions, *p* = 0.02). Administration errors decreased from 144 (41.1 per 100 infusions) to 119 (32.4 per 100 infusions, *p* = 0.12). Expired medication errors significantly reduced from 11 (3.1 per 100 infusions) to 2 (0.5 per 100 infusions, *p* = 0.02). Errors involving high‐risk medications significantly reduced from 45 (12.8 per 100 infusions) to 25 (6.8 per 100 infusions, *p* = 0.01). Errors involving continuous medications significantly reduced from 44 (12.6 per 100 infusions) to 22 (6.0 per 100 infusions, *p* = 0.005). When comparing programming type, manual programming resulted in 115 (77.2%) of administration and user documentation errors compared with 34 errors (22.8%) that occurred when auto programming was used. Of these, errors involving high‐risk medications reduced from 21 (84.0%) to 4 (16.0%) after using auto programming (OR 0.69, 95% CI [0.51, 0.93])^a^	Moderate
Khari and Pazokian 2022	Iran	Quasi‐experimental, pretest‐posttest	1 hospital emergency ward	52 emergency department nurses	Educational programme; simulation training on nurses' drug errors	The mean and standard deviation of the difference in medication errors before and after the intervention was 0.31 ± 0.55 for non‐injectables and 0.18 ± 0.61 for injectable drugs. A yearly increase in nurse working experience was associated with a decrease in medication errors of −1.73	High
Greengold et al. 2003	USA	Randomised Controlled Trial	2 hospitals (academic community hospital and a university teaching hospital)	8 nursing units caring for medical or surgical patients	Educational program; The use of “dedicated” nurses after receiving a brief review course on safe medication use	At both hospitals combined, the total error rate was 15.7% for medication nurses and 14.9% for general nurses (*p* < 0.84). Comparing hospitals, the total error rate for medication nurses at hospital B was significantly higher than it was at hospital A (19.7% vs. 11.2%; *p* < 0.04). At hospital A, there was a significantly lower error rate for medication nurses than for general nurses in the surgical units (*p* < 0.01) but no significant differences in total errors comparing nurse types in the medical units (*p* < 0.77) (OR 1.71, 95% CI [1.47, 1.98])^a^	High
Mortaro et al. 2019	Italy	Pre and post‐intervention observational study	48‐bed Geriatric unit (381‐bed secondary care hospital)	Nurses—24 Drug dispensing rounds—44 945 drugs administered to 491 patients in T0 and 994 drugs to 506 patients in T1	Low Resource Ward‐based Interventions; Implementation of combined intervention/corrective measures to reduce interruptions	A significant reduction of the raw number of interruptions (mean per round from 17.31 in T0 to 9.09 in T1, *p* = 0.01), interruptions/patient rate (from 0.78 in T0 to 0.40 in T1, *p* = 0.01), and interruptions/drugs rate (from 0.44 in T0 to 0.22 in T1, *p* = 0.01) was observed	Low
Owens et al. 2020	USA	Prospective before and after intervention study	55‐bed emergency department (community hospital)	676 medication administrations were observed in the period before bar‐code medication administration implementation and 656 after	Work‐flow Smart Technologies; Implementation of barcode medication administration	MAE rate pre‐implementation was 2.96% with “wrong dose” errors being the most common. After bar‐code medication administration implementation, the MAE rate fell to 0.76%, a relative reduction of 74.2% (Fisher exact *p* < 0.01). The average (SD) Medication Administration System—Nurses Assessment of Satisfaction score pre‐implementation was 2.60 (0.75) and improved to 2.29 (0.66) (*t* = 2.00, *p* = 0.05) 1 month post‐implementation. (OR 0.25, 95% CI [0.09, 0.68])^a^	High
Tromp et al. 2009	Netherlands	Prospective before and after intervention study	2 nursing departments of internal medicine at a University Medical Centre	132 observations were performed; 66 (at each ward). nurses—72	Protocolised Improvement Strategy; Implementation of the protocol regarding preparation and administration of intravenous drugs	At baseline, average quality scores for nurses at the two departments were 64 (intervention ward) and 67 (control ward) on a 0–100 quality scale. The pre‐test quality scores were not statistically significant for the two nursing wards (*T* = 1.36, df = 55, *p* = 0.18). After the implementation of the new protocol, nurses at the intervention ward scored better (72) than nurses at the control ward (69). The mean score at the intervention ward was significantly higher than the score of nurses in the control ward (*T* = −2.20, df = 53, *p* = 0.04)	Moderate
Schneider et al. 2006	USA	Randomised, controlled, non‐blinded study	3 community hospitals (medical–surgical units)	30 nurses (10 per site)	Educational program; Implementation of an interactive CD‐ROM program on safe medication practice	The majority of errors made were core 1 errors. The nurse‐level data showed a significant decrease in core 1 error rates between baseline and post intervention periods. Core 2 error rates were higher in the post intervention period, but the increase was not significant. Very few core 3 errors were made by either group during either period A random‐effects logistic regression model was constructed using the nurse‐level data. Core 1 errors significantly decreased during the postintervention period (OR 0.38; 95% CI, 0.19–0.74) (*p* = 0.004) (OR 2.07, 95% CI [0.95, 4.50])^a^	High
Carey et al. 2008	United Kingdom	Prospective observational	6 wards in a single hospital trust	Adult patients 27—before 29—after intervention	Educational programme; Inclusion of diabetes specialist nurse prescriber in management of diabetic patients	There was a significant reduction in the total number of errors between the pre‐intervention and intervention groups (mean reduction 21 errors) (*p* = 0.016). The median length of stay was reduced by 3 days. The total number of errors and length of stay were affected by admission category (*p* = 0.0004). There was a significant difference and reduction in the total number of errors between the pre‐intervention group (*M* = 26, SD = 35.04) and intervention group [*M* = 5.03, SD = 7.79; *t*(47) = 2.632, *p* = 0.016]	High
Ibarra‐Pérez et al. 2021	México	Retrospective descriptive study	Adult ICU of the Hospital Juárez de México (HJM)	124,229 infusion programs	Work‐flow Smart Technologies; implementation of IV smart pump/dose error reduction system—DERS (Hospira MedNet) technology	The system monitored 124,229 infusion programs and averted 36,942 deviations of the preset safe limits. Upper hard limit alerts accounted for 26.4% of pump reprogramming events. One hundred sixty‐six significant administration errors were intercepted and prevented, and IV Medication Harm Index analysis identified 83 of them as highest risk averted overdoses, with insulin accounting for 51.8% of those. The rate of compliance with the safety software during the study period was 69.8%.	Moderate
Chapuis et al. 2010	France	Prospective Pre and post‐intervention study	2 Adult medical intensive care units (a 2000‐bed university hospital)	1476 medications for 115 patients Nurses—68	Work‐flow Smart Technologies; Implementation of automated dispensing system	There was a reduced percentage of total opportunities for error in the study compared to the control unit (13.5% and 18.6%, respectively; *p* < 0.05); however, no significant difference was observed before automated dispensing system implementation (20.4% and 19.3%, respectively; not significant). Before‐and‐after comparisons in the study unit also showed a significantly reduced percentage of total opportunities for error (20.4% and 13.5%; *p* < 0.01). An analysis of detailed opportunities for error showed a significant impact of the automated dispensing system in reducing preparation errors (*p* < 0.05). Most errors caused no harm. The automated dispensing system did not reduce errors causing harm. Finally, the mean for working conditions improved from 1.0 ± 0.8 to 2.5 ± 0.8 on the four‐point Likert scale (OR 0.92, 95% CI [0.54, 1.58])^a^	High
Berdot et al. 2021	France	Multicentre, cluster, controlled, randomised study	29 adult units (4 hospitals)	178 nurses, 1346 patients, 383 medication rounds	Low Resource Ward‐based Interventions; Implementation of a ‘do not interrupt’ vest	During the intervention period, the administration error rates were 7.09% (188 OE with at least one error/2653 TOE) for the experimental group and 6.23% (210 OE with at least one error/3373 TOE) for the control group (*p* = 0.192). Identified risk factors (patient age, nurses' experience, nurses' workload, unit exposition, and interruption) were not associated with the error rate. The main error type observed for both groups was wrong dosage form. Most errors had no clinical impact for the patient, and the interruption rates were 15.04% for the experimental group and 20.75% for the control group (OR 1.15, 95% CI [0.94, 1.41])^a^	High
Ford et al. 2010	USA	Prospective before and after intervention study	Adult coronary critical care (CCU) and medical intensive care units (MICU) (University of Pittsburgh Medical Centre, Presbyterian Hospital is a level 1 regional resource trauma centre)	880 doses (402 CCU, 478 MICU) Nurses—24	Educational program includingsimulation‐based training for CCU nurses and a didactic lecture for MICU nurses.	After the simulation‐based educational intervention in the CCU, MAE rates decreased from 30.8% – 4.0% (*p* < 0.001) in the initial post‐intervention observation and were sustained in the final post‐intervention observation (30.8% – 6.2%; *p* < 0.001). The error rate in the MICU after the didactic lecture intervention was not significantly different from the baseline and increased in the final post‐intervention observation from 20.8% – 36.7% (*p* = 0.002). Mean quiz scores were significantly improved after education sessions in both ICUs (OR 0.14, 95% CI [0.06, 0.35])^a^	High
Trbovich et al. 2010	Canada	Experimental study with a repeated measures design	High‐fidelity simulated inpatient unit	24 nurses	Work‐flow Smart Technologies; Comparism of infusion pump technologies (traditional pump vs. smart pump vs. smart pump with barcode)	The nurses remedied 60% of “wrong drug” errors. This rate did not vary as a function of pump type. The nurses remedied “wrong patient” errors more often when using the barcode pump (88%) than when using the traditional pump (46%) or the smart pump (58%) (Cochran *Q* = 14.36; *p* < 0.05). The number of nurses who remedied “wrong dose hard limit” errors was higher when using the smart pump (75%) and the barcode pump (79%) than when using the traditional pump (38%) (Cochran *Q* = 12.13; *p* < 0.003). Success rates on secondary infusions were low (55.6%) and did not vary as a function of pump type.	Moderate
Straight 2008	USA	Prospective before‐and‐after study	3 healthcare institutions (acute community‐based healthcare organization)	41 nurses	Educational program including: Online continuing education module on nurses' use of the Lexi‐Comp feature of the Pyxis MedStation 2000, a point of care automated medication delivery unit (AMDU)	After training, completion of the tutorial and knowledge and use of the Lexi‐Comp feature increased by 23% and 56%, respectively. Prior to the study, 22% of the errors were categorized as administrative. One month after the study, there was a drop in administrative medication errors at both acute care facilities: 40.8% at the first and 21.5% at the second	Moderate
Young et al. 2015	USA	Prospective, pre‐post study	Rural critical access hospital	200 medical records of elderly cardiac patients (65 years of age or older)	Educational program; Effectiveness of advanced practice nurse (APN)‐managed medication reconciliation programs on medication discrepancy and patient outcomes	The chi‐square test shows that the proportion of patients with at least one medication discrepancy reduced from 94% before the intervention to 81% after the intervention (*χ* ^2^1 = 7.726, *n* = 200, *p* = 0.005). The mean number of medication discrepancies for each patient was reduced from 8.09 ± 6.75 in the pre‐intervention group to 4.32 ± 5.66 in the post‐intervention group (*p* = 0.005). The average number of unintentional medication discrepancies per patient reduced from 5.09 ± 4.60 in the pre‐intervention group to 0.30 ± 1.904 in the post‐intervention group (*F* = 52.941, *p* = 0.000). The intervention had more of an effect on reducing the discrepancies caused by incorrect addition (1.80 ± 1.74 in the pre‐intervention group, 0.20 ± 1.46 in the post‐intervention group; *p* = 0.00) and omission (1.76 ± 3.20 in the pre‐intervention group, 0.01 ± 0.10 in the post‐intervention group; *p* = 0.00), which were also the most common types of medication errors There was no difference in the intentional medication discrepancy caused by adding new medications (1.77 ± 1.75 in the pre‐intervention group, 1.75 ± 1.96 in the post‐intervention group; *p* = 0.886)	Moderate
FitzHenry et al. 2007	USA	Retrospective/descriptive analysis	Adult patient in sub‐acute, acute, and critical care units (a 658‐bed tertiary care academic hospital)	190 adult patient charts	Work‐flow Smart Technologies; Measurement of the degree to which computerized physician order entry (CPOE) systems medication orders matched actual dose administration times	Covered 1307 inpatient administration‐days, with median audited days per patient of 6.9 (IQR 4.5–10.0). By design, the study truncated auditing of 59 patient records after hospital day 10. The 1502 audited medication orders involved 6019 medication administration opportunities. Dose omissions occurred in 756 of 6019 (12.6%) audited administration opportunities; only 313 of the omissions (5.2% of opportunities) were unexplained. Wrong doses and unexpected doses occurred for 0.1% and 0.7% of opportunities, respectively. Median lag from expected first dose to actual charted administration time was 27 min (IQR 0–127). Nursing staff shifted from ordered to alternate administration schedules for 10.7% of regularly scheduled recurring medication orders. Chart review identified reasons for dose omissions, delays, and dose shifting	Moderate

^a^ Highlight OR calculated from baseline data presented in the respective study.

### 3.1 Study Characteristics

Selected studies were conducted in seven countries, with the majority (*n* = 10) conducted in the USA (Booth et al. 2024; Nash et al. 2005; Skog et al. 2022; Greengold et al. 2003; Owens et al. 2020; Schneider et al. 2006; Ford et al. 2010; Straight 2008; Young et al. 2015; FitzHenry et al. 2007), four from the Netherlands (van der Veen et al. 2020; Schimmel et al. 2011; Verweij et al. 2014; Tromp et al. 2009), three from Australia (Johnson et al. 2017; Westbrook et al. 2020; Redley and Botti 2013), two from France (Chapuis et al. 2010; Berdot et al. 2021), and one each from the United Kingdom, Sweden, Mexico, South Africa, Iran, Italy, and Canada (Carey et al. 2008; Bergqvist et al. 2009; Ibarra‐Pérez et al. 2021; Allison Rout et al. 2023; Khari and Pazokian 2022; Mortaro et al. 2019; Trbovich et al. 2010).

Studies were conducted in various clinical areas; 17 studies were conducted in the wards, five in intensive care units (ICU), three in emergency departments, and one in a high‐fidelity simulated inpatient unit.

Four studies were Randomised Control Trials (RCTs) and 22 were quasi‐experimental studies; of these, one was a case–control study and another was a cross‐sectional study, four were retrospective cohort and descriptive studies, and 16 studies were prospective before and after studies (see Table 1).

### 3.2 Risk of Bias Assessment

All included studies clearly stated the study objectives, study population, and interventions. Study quality assessment using the JBI tool (Cross Sectional Studies, Cohort Studies, Case Control Studies, Quasi‐Experimental studies, and Randomised Control Trials) showed that included studies were of high (*n* = 12), medium (*n* = 12), and low (*n* = 2) quality (see Table 1 and Supporting Information); thus, evidence gathered from studies could influence and inform practice. All studies had a likelihood of bias, as there is at least one missing element of the study quality indicators on the critical appraisal checklists. The most common source of risk of bias was a lack of blinding to the outcome due to the study design (direct observation). Furthermore, the inherent design of non‐randomised controlled studies precludes the use of randomisation and assessor blinding.

### 3.3 Interventions

Five different types of interventions were identified: educational programme (*n* = 8), workflow smart technologies (includes automated, semi‐automated, and digital technologies, *n* = 8), protocolised improvement strategy (*n* = 1), low resource ward‐based interventions (local low cost, easy to implement ward‐based response system, *n* = 7), and electronic medication management (*n* = 2). Three studies used multicomponent/bundle interventions (Booth et al. 2024; Johnson et al. 2017; Allison Rout et al. 2023), while the others focused on a single intervention. Table 1 shows the description of the intervention and outcome for each study.

### 3.4 Outcomes

The studies looked at the effectiveness of interventions implemented by nurses to reduce MAEs in hospitalised acute adults' settings. Nine studies were conducted across more than one hospital; eight studies were conducted in different units within a single site, eight studies were conducted in a single hospital unit, and one study was conducted in a high‐fidelity simulated inpatient unit for nurses.

### 3.5 Meta‐Analysis

**FIGURE 2:**
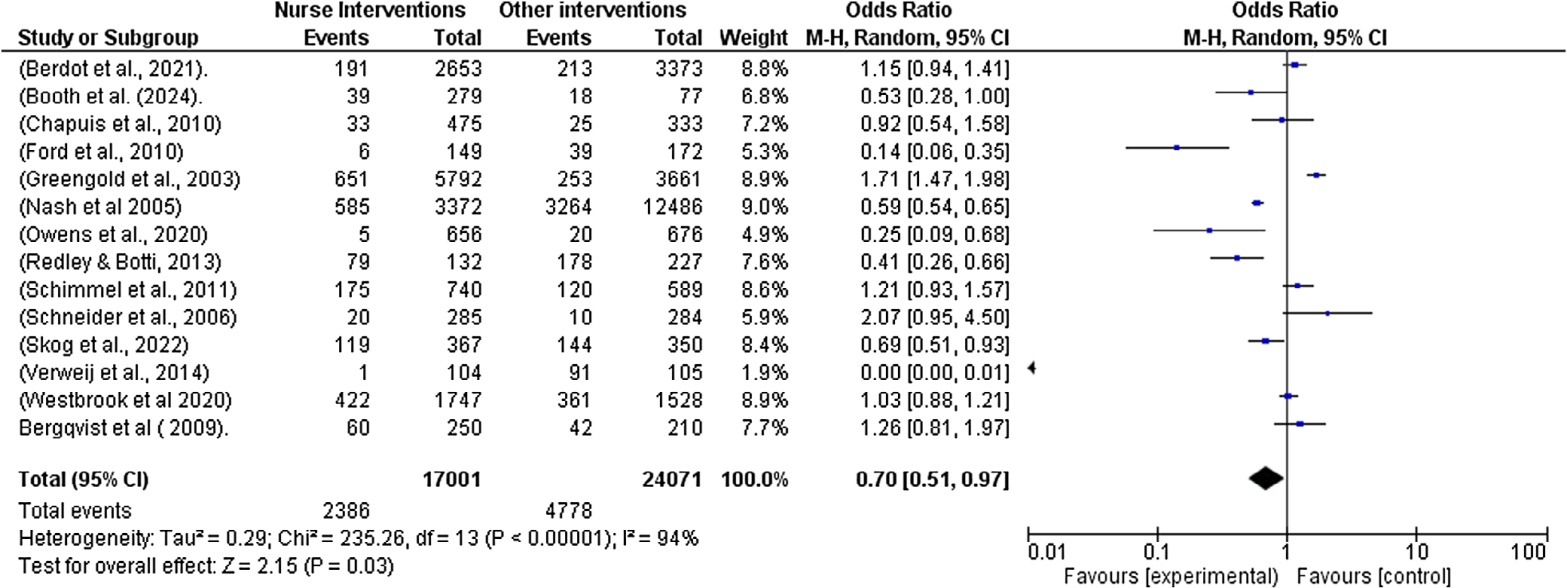
Forest Plot all Interventions. [Colour figure can be viewed at wileyonlinelibrary.com]

Fourteen studies (Booth et al. 2024; Nash et al. 2005; Skog et al. 2022; Greengold et al. 2003; Owens et al. 2020; Schneider et al. 2006; Ford et al. 2010; Schimmel et al. 2011; Verweij et al. 2014; Westbrook et al. 2020; Redley and Botti 2013; Chapuis et al. 2010; Berdot et al. 2021; Bergqvist et al. 2009) with 35,046 participants were included in the meta‐analysis. The results show a significant reduction in MAEs while using interventions implemented by nurses compared to the non‐intervention group (*Z* = 2.15 (*p* = 0.03); odds ratio (OR) and 95% confidence intervals (CI) 0.70 [0.51, 0.97]). See Figure 2 forest plot for all interventions.

A high level of heterogeneity was observed (*I* ^2^ = 94%). This may be attributed to the following factors. Firstly, the studies included in the meta‐analysis employed a variety of methodologies, including randomised controlled trials (RCTs), observational studies, cohort studies, pre‐and‐post designs, and cross‐sectional analyses. Incorporating both randomised and non‐randomised studies may introduce bias, as RCTs are generally regarded as having a lower risk of bias compared to non‐RCTs. Consequently, combining these study types could increase the likelihood of confounding factors affecting the results. Secondly, this may be attributed to different clinical contexts in which the studies were conducted, and thirdly, some of the studies' odds ratio statistical data used in the meta‐analysis was calculated from raw data provided in the papers.

Given the variability in methodologies among the studies included in the meta‐analysis, a random effects model was employed. Although the substantial heterogeneity warrants cautious interpretation of the pooled effect estimates, the findings remain valuable as individual studies consistently indicate significant reductions in medication administration errors. In addition, subgroup analysis was conducted to identify potential sources of variability, yielding positive results. See Figure 2 forest plot for all interventions.

**FIGURE 3:**
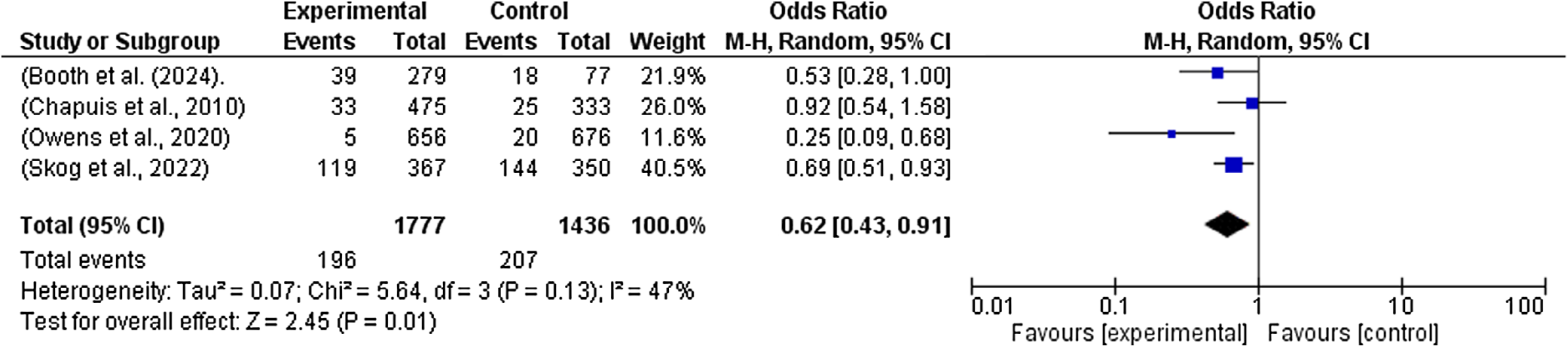
Workflow Smart Technologies. [Colour figure can be viewed at wileyonlinelibrary.com]

**FIGURE 4:**
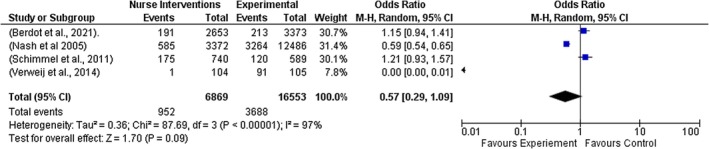
Low Resource Ward‐Based Interventions. [Colour figure can be viewed at wileyonlinelibrary.com]

**FIGURE 5:**
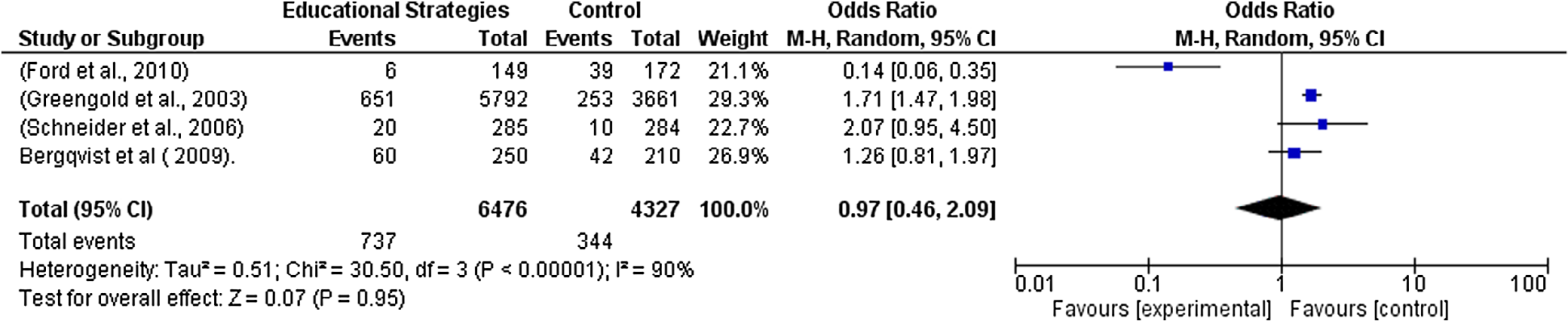
Educational Programme. [Colour figure can be viewed at wileyonlinelibrary.com]

**FIGURE 6:**
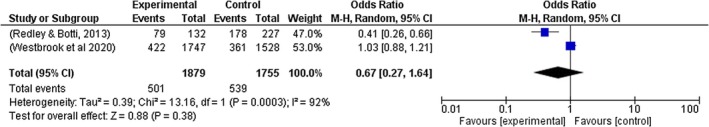
Electronic Medication Management. [Colour figure can be viewed at wileyonlinelibrary.com]

A sub‐group analysis was performed to account for differences in effect estimates depending on the types of interventions implemented by nurses. The following results were observed: Electronic medication management system (*Z* = 0.88, *p* = 0.38, OR = 0.67 [95% CI = 0.27, 1.64], *I* ^2^ = 92%); Educational intervention and strategies (*Z* = 0.07, *p* = 0.95, OR = 0.97 [95% CI = 0.46, 2.09], *I* ^2^ = 90%); Work‐flow software strategies (Z = 2.45, *p* = 0.01, OR = 0.62 [95% CI = 0.43, 0.91], *I* ^2^ = 47%); and Low resource ward‐based interventions (Z = 1.70, *p* = 0.09, OR = 0.57 [95% CI = 0.29, 1.09], *I* ^2^ = 97%). See Figures 3, 4, 5, 6.

### 3.6 Publication Bias

**FIGURE 7:**
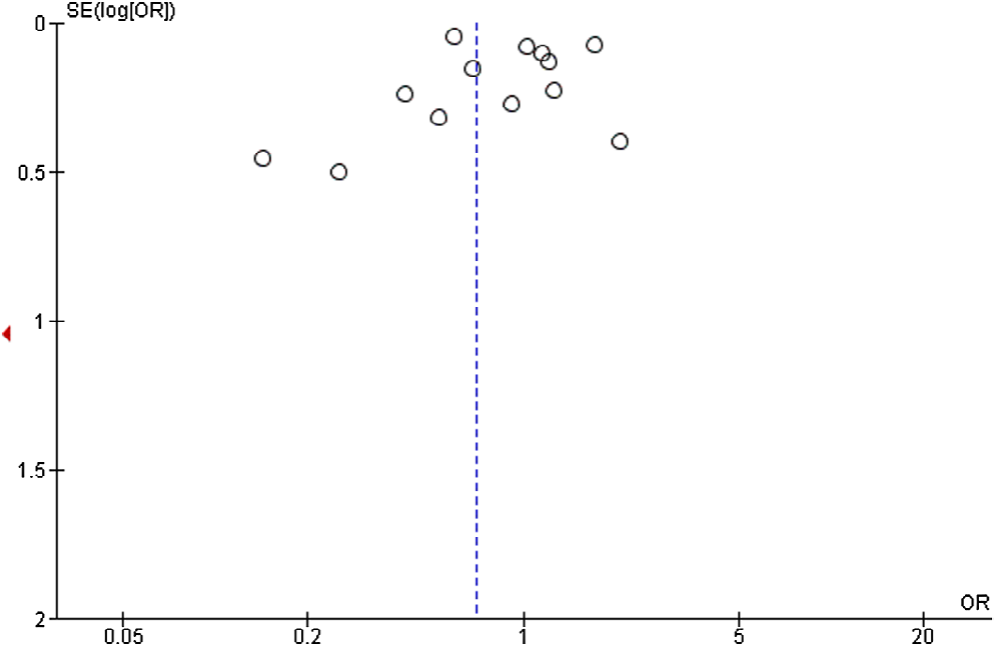
Funnel Plot. [Colour figure can be viewed at wileyonlinelibrary.com]

A check for publication bias was assessed visually using a funnel plot developed in RevMan 5.4 software. The funnel plot was asymmetric, which reveals a possible risk of publication bias. One study (Verweij et al. 2014) was not published on the plot, and this indicates a negative or non‐significant result from the missing study (Figure 7).

### 3.7 Narrative Synthesis

#### 3.7.1 Work‐Flow Smart Technologies

Four studies reported using barcode‐assisted medication, computerised physician order entry (CPOE), and IV smart pumps (van der Veen et al. 2020; FitzHenry et al. 2007; Ibarra‐Pérez et al. 2021; Trbovich et al. 2010).

In a study by van der Veen et al. (2020), the use of barcode‐assisted medication administration with over 5000 (5793) medication administrations among 1230 patients given by 272 nurses was observed. In 3633 (62.7%) of the administrations, one or more workarounds were observed, and several potential risk factors associated with workarounds performed by nurses that could be used to target future improvement efforts in barcode‐assisted medication administration were identified.

The study by Ibarra‐Pérez et al. (2021) reported that IV smart pump and dose error reduction system (DERS) monitored 124,229 infusion programs and averted 36,942 deviations of the present safe limits, the upper hard limit alerts accounted for 26.4% of pump reprogramming events. The study also reported that 166 significant administration errors were intercepted and prevented, and IV Medication Harm Index analysis identified 83 of them as the highest risk averted overdoses with insulin accounting for 51.8%.

On the other hand, the findings of Trbovich et al. (2010) study reveal that 24 nurses assessed in a high‐fidelity simulated inpatient unit remedied 60% of “wrong drug” errors, but this rate did not vary as a function of pump type. “Wrong patient” errors were remedied more often when using the barcode pump (88%) than when using the traditional pump (46%) or the smart pump (58%) (Cochran *Q* = 14.36; *p* < 0.05). Nonetheless, the number of nurses who remedied “wrong dose hard limit” errors was higher when using the smart pump (75%) and the barcode pump (79%) than when using the traditional pump (38%) (Cochran *Q* = 12.13; *p* < 0.003).

Finally, FitzHenry et al. (2007) reported that dose omissions occurred in 756 of 6019 (12.6%) audited administration opportunities using computerised provider order entry (CPOE); only 313 of the omissions (5.2% of opportunities) were unexplained. Wrong doses and unexpected doses occurred for 0.1% and 0.7% of opportunities, respectively.

#### 3.7.2 Low Resource Ward‐Based Interventions

Three studies (Johnson et al. 2017; Mortaro et al. 2019; Allison Rout et al. 2023) employed a cluster RCT design to test the intervention of nurse‐initiated recall cards and cross‐checking medication charts during nursing handover, evaluation of the effectiveness of implementing combined intervention and corrective measures to reduce interruptions during medication preparation and administration, and observation of double‐checking processes and infusion labelling practices, respectively.

Johnson et al. (2017) in their study identified no statistically significant difference in either of the omission rates between the control and intervention arms (omission rate without documentation: *p* = 0.25; omission rate of all omissions: *p* = 0.40) of 584 patients observed. Conversely, Mortaro et al. (2019) in their study identified a significant reduction in the raw number of interruptions (mean per round from 17.31 in T0 to 9.09 in T1, *p* = 0.01), interruptions/patient rate (from 0.78 in T0 to 0.40 in T1, *p* = 0.01), and interruptions/drug rate (from 0.44 in T0 to 0.22 in T1, *p* = 0.01) observed in 945 drugs administered to 491 patients in T0 and 994 drugs to 506 patients in T1.

Furthermore, Allison Rout et al. (2023) reported that of the 223 Carbapenem infusion administrations to 23 patients observed, adherence to the scheduled time occurred in 34.9% of administrations, 5.4% of doses was not given, and an incorrect dose was given in 1.4% of administrations.

#### 3.7.3 Educational Programme

Four studies assessed the impact of educational programmes and interventions on reducing MAES. Two were purely educational interventions: continuing education (Straight 2008) and simulation training (Khari and Pazokian 2022). The other two reported the impact of specialist nurses on MAEs, namely diabetes specialist nurse (Carey et al. 2008) and advanced practice nurse (Young et al. 2015).

In the study by Straight (2008), the knowledge and use of the Lexi‐Comp feature (a point of care automated medication delivery unit) increased by 23% and 56%, respectively. Prior to the study, 22% of the errors were categorized as administrative. One month after the study, there was a drop in administrative medication errors at both acute care facilities: 40.8% at the first and 21.5% at the second. Moreover, in the study by Khari and Pazokian (2022), the mean and standard deviation of the difference in medication errors before and after the intervention was 0.31 ± 0.55 for non‐injectable and 0.18 ± 0.61 for injectable drugs.

On the other hand, Carey et al. (2008) reported a significant reduction in the total number of errors between the pre‐intervention and intervention group (mean reduction 21 errors, *p* = 0.016). Similarly, in the study by Young et al. (2015), the chi‐square test shows that the proportion of patients with at least one medication discrepancy reduced from 94% before the intervention to 81% after the intervention (*χ* ^2^1 = 7.726, *n* = 200, *p* = 0.005). The average number of unintentional medication discrepancies per patient reduced from 5.09 ± 4.60 in the pre‐intervention group to 0.30 ± 1.904 in the post‐intervention group (*F* = 52.941, *p* = 0.000). The intervention had more of an effect on reducing the discrepancies caused by incorrect addition (1.80 ± 1.74 in the pre‐intervention group, 0.20 ± 1.46 in the post‐intervention group; *p* = 0.00) and omission (1.76 ± 3.20 in the pre‐intervention group, 0.01 ± 0.10 in the post‐intervention group; *p* = 0.00), which were also the most common types of medication errors.

#### 3.7.4 Protocolised Improvement Strategy

In a study by Tromp et al. (2009), a new protocol for preparation and administration of intravenous drugs was tested. At baseline, average quality scores for nurses at the two departments were 64 (intervention ward) and 67 (control ward) on a 0–100 quality scale. After the intervention, nurses in the intervention ward scored better (72) than nurses in the control ward (69). The mean score in the intervention ward was significantly higher than the score of nurses in the control ward (*T* = −2.20, df = 53, *p* = 0.04).

#### 3.7.5 Electronic Medication Management

The two studies in this category were included in the meta‐analysis (Westbrook et al. 2020; Redley and Botti 2013) OR; 0.67 [95% CI; 0.27, 1.64, *I* ^2^ = 92%; *Z* = 0.88, *p* = 0.38]. While the results show a reduction in MAEs by implementing the EMMs, the subgroup analysis results were not statistically significant. See Figure 2 forest plot for all interventions and Figure 6: Electronic Medication Management for subgroup analyses.

## 4 Discussion

This review provides an update of medication administration error interventions implemented by nurses in current practice to reduce errors in adult patients admitted to hospital. Studies included in this review utilised various designs and terminologies related to MAEs and reporting standards. Studies reported medications ranging from tablets, intravenous infusions, and injections, which provide wider coverage to generalise the results of this review. Similarly, studies were conducted in different adult acute and hospital areas, further enhancing the evidence's generalisation. The report on different study designs and variations to different administration error types from the studies made comparing study findings challenging. Nonetheless, the majority of the studies report findings from various clinical areas, enhancing the applicability of the evidence.

From the review findings, there were variations in the error rates from different hospitals as well as different areas of the same hospital, which suggests that organisational factors like patient population and nurse‐to‐patient ratio could be determinants of MAEs (Jessurun et al. 2023). Hence, these determinants should have a targeted focus of interventions to improve patient safety.

The most observed intervention implemented by nurses among the five themes in reducing MAEs in this review was workflow smart technologies. Most of the studies were conducted after 2020, acknowledging the increased use of technology within healthcare and among nurses. Alamer and Alanazi (2023) in their review reported results that support our findings; smart technologies decrease errors such as incorrect rate and dose. However, they highlighted that these technologies' efficiency is reliant on the nurses' compliance. In the ward setting, compliance may be influenced by the complexity of smart infusion pumps and the tendency to ignore soft alerts (Alamer and Alanazi 2023).

Thompson et al. (2018) also highlight the findings of our review by reporting that barcode medication administration decreased MAEs by 43.5%. However, Mulac, Taxis, et al. (2021) in their study were concerned that barcode technology may cause some task deviations during dispensing and administration due to technological factors and environmental factors (room location). Hence, this review recommends that barcode interventions are adapted to work systems such as policies and technology to enhance patient safety. Similarly, Manias et al. (2020) also reported in their review that MAEs were reduced by CPOE and the use of an automated drug distribution system as single interventions. Nonetheless, CPOE has been associated with workflow issues and more work for health professionals (Elshayib and Pawola 2020). Concerning high‐risk medications like heparin, nurses have expressed satisfactory knowledge regarding route, regulation, and patient assessment (Pierobon et al. 2022). As this review has revealed the importance of heparin calculators, we also recommend good numeracy knowledge for all nurses to enhance their confidence and competence as well as improve patient safety.

The second most common theme of the review revealed that educational interventions have potential in reducing MAEs compared to usual care. Education activities observed in this review include online module (Straight 2008); simulation‐based training (Ford et al. 2010; Khari and Pazokian 2022); interactive CD‐ROM programme (Schneider et al. 2006); pharmacology training (Bergqvist et al. 2009); and use of dedicated medication nurse (Greengold et al. 2003).

Farzi et al. (2020) suggest that an inexpensive educational strategy like blended learning can improve nursing staff performance and reduce medication errors. Mohanna et al. (2022) in their review identified that different educational approaches for nurses were found to be effective in reducing medication preparation and medication administration errors. However, they also noted several limitations despite the reported positive effects such as long‐term effects not investigated, as there was a potential risk that nurses would return to pre‐intervention practices over time; this also limited the generalisability of the results. Similarly, Ciapponi et al. (2021) in their review highlighted that the grade of evidence for education on administration (nurses) had very low certainty with very little confidence in the effect estimate [OR 1.64 (95% CI 0.88 to 3.08)], which signified that the true effect was likely to be substantially different from the estimate of effect.

The third theme of the review indicated that low‐resource ward‐based interventions may contribute to a reduction in medication administration errors (MAEs), although the meta‐analysis showed substantial heterogeneity among studies. These interventions included strategies such as the use of drug round tabards—aprons worn by nurses during medication administration to minimize interruptions and promote patient safety (Verweij et al. 2014; Berdot et al. 2021), implementation of medication safety reporting systems (Nash et al. 2005), adoption of a second medication cart filling method (Schimmel et al. 2011), utilization of nurse recall cards (Johnson et al. 2017), and combined approaches aimed at reducing interruptions and errors, including double‐checking, documentation, and labeling (Mortaro et al. 2019; Allison Rout et al. 2023).

Koyama et al. (2020) highlighted in their review that double‐checking, as implemented by nurses, is not an effective intervention for reducing medication administration errors (MAEs). This may be attributed to insufficient reporting on adherence levels and the lack of a clear definition for double‐checking. Similarly, Mazzitelli et al. (2018) reported findings consistent with our review, noting that while drug round tabards were effective in minimizing distractions and interruptions, this did not consistently translate to a reduction in MAEs. Based on these findings, we recommend that reliance on this intervention alone should be avoided for reducing MAEs on wards. In conjunction with the use of drug round tabards, fostering effective teamwork and displaying ‘do not disturb’ signage in all work areas are advised to further mitigate interruptions.

The fourth theme, electronic medication management, was another convincing finding throughout the results of the two studies (Westbrook et al. 2020; Redley and Botti 2013) that measured their effectiveness in reducing medication administration errors. Both studies reported a modest but significant reduction in the overall MAE rate and suggest there are differences in the types of medication errors that were reported in association with the introduction of the electronic medication management system compared to pen and paper systems. Both studies were conducted in Australia, were multi‐centre studies, and scored high quality respectively.

The implementation of electronic medication systems (EMS) is intended to decrease medication errors; however, findings on the effectiveness of these systems are mixed, according to Gates et al. (2021). Their study, which assessed changes in medication error rates after EMS adoption, found that although EMS has been widely introduced in hospitals around the world, the evidence regarding their impact on reducing errors varies widely. Stolic et al. (2023) also investigated if medication error rates were reduced during nursing administration when incorporating electronic medical administration records into medication management. However, their findings were mixed, as some studies reported positive findings and a reduction in medication errors, while other studies reported no reduction in medication errors or the introduction of new types of errors.

### 4.1 Strengths and Limitations

The review was preceded by a protocol registered with PROSPERO which outlined the inclusion and exclusion criteria, search strategy, extraction and critical assessment tools with no deviations throughout its conduct. A comprehensive search was conducted beyond six database including grey literature which minimised bias and strengthened the synthesis of the review evidence. Studies were conducted in different adult acute and hospitalised areas, multiple clinical areas, countries and regions further enhancing evidence's generalisability.

However, several limitations are notable. First, most studies employed different designs and terminologies related to medication administration errors and reporting unstandardised MAEs; this made comparing study findings challenging. In addition, the exclusion of studies published in languages other than English may have reduced the likelihood of retrieving important studies conducted in countries where English is not a common language, which could impact the external validity of the review. Nonetheless, the overall risk of bias in the included studies ranges from low to moderate, which enhances the quality of evidence generated.

### 4.2 Implications to Practice

The findings from this systematic review and meta‐analysis highlight key strategies to reduce medication administration errors (MAEs) in hospitalised acute adult patients. It emphasises evidence‐based interventions, education, improved communication, technology adoption, a strong safety culture, adequate staffing, standardised policies, and continuous quality improvement.

Integrating these measures is considered valuable to enhance patient safety and nursing care through prioritising interventions with demonstrated effectiveness. Ongoing education and competency‐based training programs for nurses should focus on medication safety principles, error prevention strategies, and the use of technology‐assisted interventions while encouraging simulation‐based training, which has been shown to be beneficial in helping nurses recognise and respond to potential medication errors in a controlled environment. Effective communication and interdisciplinary collaboration between nurses and the clinical team using health information technologies are required to minimise medication errors and improve patient outcomes; therefore, hospitals should invest in user‐friendly, well‐integrated digital solutions that support nurses in medication administration.

A strong medication safety culture, supported by non‐punitive error reporting, is important in fostering a learning environment where systemic issues could be addressed to prevent future occurrences. Addressing workload and staffing challenges ensures adequate nurse‐to‐patient ratios, reducing fatigue‐related errors. Policy standardisation and continuous quality improvement, including audits and real‐time feedback, are essential for sustaining safe practices across healthcare settings. Health Institutions are thereby encouraged to establish robust monitoring systems, such as medication error audits, root cause analyses, and real‐time feedback mechanisms, to assess the effectiveness of interventions and drive continuous improvements in medication safety.

## 5 Conclusion

The issues around MAEs remain an area of concern for hospitalised patients. This systematic review and meta‐analysis demonstrate overall that interventions implemented by nurses have the potential to reduce MAEs compared to usual care. However, subgroup analysis identified that workflow smart technologies had superiority when considering the effectiveness of those interventions versus usual care. It is important to have improved commitment from nurses to adopt evidence‐based interventions for the safe administration of medications in clinical practice, with continuous monitoring and evaluation of their effectiveness as key for ensuring patient safety. In addition, there is also a need to have proper randomised controlled trials involving a bundle of interventions rather than a single intervention to provide robust evidence of the effectiveness of interventions implemented by nurses.

## Disclosure

The first author conducted this review through a NIHR/NHSE–East Midlands Clinical Academic Internship.

## Conflicts of Interest

The authors declare no conflicts of interest.

## Supporting information


**Appendix S1:** jocn70109‐sup‐0001‐AppendixS1.docx.

**Appendix S2:** jocn70109‐sup‐0002‐AppendixS2.docx.

## Data Availability

The data that supports the findings of this study is available in the Supporting Information of this article.
