# Supplementary material for: Assessing the Effectiveness of Interventions Implemented by Nurses to Reduce Medication Administration Errors in Hospitalised Acute Adult Patient Settings: Systematic Review and Meta‐Analysis
Source: J Clin Nurs. 2025 Oct 1;35(3):1104–24. doi: 10.1111/jocn.70109 (PMC12862564; doi:10.1111/jocn.70109)
Supplement: Supplementary file 2 — Appendix S2: jocn70109‐sup‐0002‐AppendixS2.docx. [file JOCN-35-1104-s001.docx]

Full Title: Assessing the effectiveness of interventions implemented by nurses to reduce medication administration errors in hospitalised acute adult patient settings; systematic review and meta-analysis.

Short Title: Medication administration errors

**Supplementary data/ Supporting information**

| **Population** | **context** | **Intervention** | **Comparisons** | **Outcome** | **Type of Medication Administration Error** |
| --- | --- | --- | --- | --- | --- |
| Adults | inpatients | Education | Standard care | Reduction in rate of MAE | Omission or significantly delayed dose |
| Nurses | Ward | Training | No intervention | Number of incidents | Incorrect day/time |
|  | Acute | Service redesign |  | Improvements to care | Incorrect dose |
|  | Hospital | Technology |  | Error reduction | Incorrect frequency |
|  | Admission | Communication |  | Patient safety | Incorrect drug |
|  | unit | Prevention |  | evaluation |  |
|  |  | Knowledge |  | Harm reduction |  |
|  |  | Calculation |  | Error rate |  |
|  |  | Double-checking |  |  |  |
|  |  | Staffing level |  |  |  |
|  |  |  |  |  |  |

**Search Strategy**

**Database Search term Results:**

CINAHL SEARCH 07/03/2024

| **#** | **Query** | **Results** |
| --- | --- | --- |
| S12 | S8 AND S11 | 79 |
| S11 | S1 AND S4 AND S9 AND S10 | 57,740 |
| S10 | interventions or strategies or best practices or treatment or therapy or program or management | 3,441,654 |
| S9 | S5 OR S6 OR S7 | 1,702,725 |
| S8 | medication administration errors | 769 |
| S7 | adverse drug reactions or adverse drug effects or adverse drug events or side effect or adverse effect or adverse reaction | 608,521 |
| S6 | medication or drugs or medicine or pharmacological therapy | 1,371,981 |
| S5 | medication errors or drug errors or medication administration errors or drug administration errors | 17,652 |
| S4 | S2 OR S3 | 685,572 |
| S3 | (MM "Acute Care") OR "acute care" | 26,548 |
| S2 | hospital or acute setting or inpatient or ward | 677,841 |
| S1 | adults or adult or aged or elderly | 2,199,860 |

Cochrane Search 07/03/2024

ID Search

#1 MeSH descriptor: [Adult] explode all trees

#2 (elderly):ti,ab,kw (Word variations have been searched)

#3 #1 or #2

#4 MeSH descriptor: [Inpatients] explode all trees

#5 (hospitalization):ti,ab,kw (Word variations have been searched)

#6 ("Ward"):ti,ab,kw (Word variations have been searched)

#7 (acute setting):ti,ab,kw (Word variations have been searched)

#8 (acute):ti,ab,kw (Word variations have been searched)

#9 #4 or #5 or #6 or #7 or #8

#10 MeSH descriptor: [Medication Errors] explode all trees

#11 (drug errors):ti,ab,kw (Word variations have been searched)

#12 (medicine):ti,ab,kw (Word variations have been searched)

#13 (medication):ti,ab,kw (Word variations have been searched)

#14 (pharmacological therapy):ti,ab,kw (Word variations have been searched)

#15 ("adverse drug effect"):ti,ab,kw (Word variations have been searched)

#16 ("adverse drug reaction"):ti,ab,kw (Word variations have been searched)

#17 ("adverse drug event"):ti,ab,kw (Word variations have been searched)

#18 ("adverse effect"):ti,ab,kw (Word variations have been searched)

#19 (medication administation error):ti,ab,kw (Word variations have been searched)

#20 #10 or #11 or #12 or #13 or #14 or #15 or #16 or #17 or #18

#21 (intervention):ti,ab,kw (Word variations have been searched)

#22 (strategies):ti,ab,kw (Word variations have been searched)

#23 (best practices):ti,ab,kw (Word variations have been searched)

#24 (program):ti,ab,kw (Word variations have been searched)

#25 (management):ti,ab,kw (Word variations have been searched)

#26 #21 or #22 or #23 or #24 or #25

#27 #3 and #9 and #19 and #20 and #26

Embase Search 07/03/2024

1 adult.m_titl. 256691

2 adult.mp. or *adult/ or *young adult/ 10130840

3 *hospitalization/ or hospitalisation.mp. 82942

4 inpatient.mp. or *hospital patient/ 215099

5 acute setting.mp. 5094

6 ward.mp. or *ward/ 319189

7 1 or 2 10130840

8 3 or 4 or 5 or 6 583518

9 medication errors.mp. or *medication error/ 15805

10 *medication error/ or drug errors.mp. 9647

11 adverse drug reactions.mp. or *adverse drug reaction/ 146127

12 *adverse drug reaction/ or *drug effect/ or adverse drug effect*.mp. 179565

13 adverse reaction.mp. or *adverse event/ 30649

14 medication administration error*.mp. 593

15 *medication error/ or administration error.mp. 9499

16 14 or 15 9733

17 9 or 10 or 11 or 12 or 13 246573

18 intervention*.mp. 2034991

19 strateg*.mp. 2019278

20 best practice*.mp. 59557

21 program*.mp. 1842969

22 18 or 19 or 20 or 21 5239102

23 7 and 8 and 17 and 22 992

24 17 and 23 992

25 nurses.mp. or *nurse/ 295641

26 24 and 25 103

Clinicaltrial.gov searches 07/03/2024

[Search for: Other terms: medication administration error AND hospital, nursing intervention, Adult (18 - 64), Older adult (65+), Interventional studies | Card Results | ClinicalTrials.gov](https://www.clinicaltrials.gov/search?term=medication%20administration%20error%20AND%20hospital&intr=nursing%20intervention&aggFilters=ages:adult%20older,studyType:int&limit=100&page=1) – 144

[https://www.clinicaltrials.gov/search?id=AREA[NCTId](NCT03541421](https://www.clinicaltrials.gov/search?id=AREA%5bNCTId%5d(NCT03541421) OR NCT03430336 OR NCT03062852 OR NCT02816086 OR NCT01291966) - 5

Web of Science Search

#5 AND #4 AND #3 AND #2 AND #1

[455](https://www-webofscience-com.nottingham.idm.oclc.org/wos/woscc/summary/acc59175-0c3a-4935-9d42-dc3901056971-d286a80a/relevance/1)

((((ALL=(interventions )) OR ALL=(strategies)) OR ALL=(program)) OR ALL=(best practices)) OR ALL=(management)

[15,376,223](https://www-webofscience-com.nottingham.idm.oclc.org/wos/woscc/summary/7f36de58-03fc-4ad7-b676-5121e6eeb9c7-d286a661/relevance/1)

ALL=(medication administration errors)

[4,119](https://www-webofscience-com.nottingham.idm.oclc.org/wos/woscc/summary/b08e5e08-8d4d-441f-84fa-fc2cc2602ae8-d286a057/relevance/1)

(((((((((ALL=(medication errors)) OR ALL=(drug errors)) OR ALL=(medication administration errors)) OR ALL=(medication)) OR ALL=(drug)) OR ALL=(medicine)) OR ALL=(pharmacological therapy)) OR ALL=(adverse drug reactions )) OR ALL=(adverse drug events )) OR ALL=(side effects)

[20,099,638](https://www-webofscience-com.nottingham.idm.oclc.org/wos/woscc/summary/48b54f2a-f935-4761-a64d-b08d6aff0ab4-d2869acb/relevance/1)

(((((ALL=(hospitalisation)) OR ALL=(hospital)) OR ALL=(acute setting )) OR ALL=(inpatient)) OR ALL=(ward)) OR ALL=(acute care)

[10,555,432](https://www-webofscience-com.nottingham.idm.oclc.org/wos/woscc/summary/25986823-6a66-4bf6-a9f8-53a5b6da4ed3-d286877e/relevance/1)

((ALL=(adult)) OR ALL=(aged)) OR ALL=(elderly)

[5,937,160](https://www-webofscience-com.nottingham.idm.oclc.org/wos/woscc/summary/728f22e4-c2cb-4fbc-9331-5c71647acf8c-d2867b03/relevance/1)

Medline Search

| 1. | adult*.mp. or exp Adult/ or exp Young Adult/ |
| --- | --- |
| 2. | (adult* or "grown-ups" or "mature person" or "older adults").mp. [mp=title, book title, abstract, original title, name of substance word, subject heading word, floating sub-heading word, keyword heading word, organism supplementary concept word, protocol supplementary concept word, rare disease supplementary concept word, unique identifier, synonyms, population supplementary concept word, anatomy supplementary concept word] |
| 3. | exp Hospitalization/ or hospitalization*.mp. |
| 4. | Inpatients/ or inpatient*.mp. |
| 5. | "hospital patient".mp. |
| 6. | "acute setting".mp. |
| 7. | ward*.mp. |
| 8. | 1 or 2 |
| 9. | 3 or 4 or 5 or 6 or 7 |
| 10. | "medication error".mp. or exp Medication Errors/ |
| 11. | exp Medication Errors/ or "drug errors".mp. |
| 12. | "adverse drug reactions".mp. or exp "Drug-Related Side Effects and Adverse Reactions"/ |
| 13. | ("adverse drug reaction" or "drug effect" or "adverse drug effect").mp. [mp=title, book title, abstract, original title, name of substance word, subject heading word, floating sub-heading word, keyword heading word, organism supplementary concept word, protocol supplementary concept word, rare disease supplementary concept word, unique identifier, synonyms, population supplementary concept word, anatomy supplementary concept word] |
| 14. | "adverse reaction".mp. |
| 15. | "adverse event".mp. |
| 16. | exp Nurses/ or exp Medication Errors/ or exp Nursing Staff, Hospital/ or "medication administration error".mp. or exp Pharmaceutical Preparations/ or exp Medication Systems, Hospital/ or exp Patient Safety/ |
| 17. | ("medication error" or "administration error").mp. [mp=title, book title, abstract, original title, name of substance word, subject heading word, floating sub-heading word, keyword heading word, organism supplementary concept word, protocol supplementary concept word, rare disease supplementary concept word, unique identifier, synonyms, population supplementary concept word, anatomy supplementary concept word] |
| 18. | 16 or 17 |
| 19. | 10 or 11 or 12 or 13 or 14 or 15 |
| 20. | intervention*.mp. |
| 21. | strateg*.mp. |
| 22. | "best practice".mp. |
| 23. | program*.mp. |
| 24. | 20 or 21 or 22 or 23 |
| 25. | 8 and 9 and 19 and 24 |
| 26. | 19 and 25 |
| 27. | nurses*.mp. or exp Nurses/ |
| 28. | exp Nurses/ or nurse*.mp. |
| 29. | 26 and 27 |
| 30. | 26 and 28 |
| 31. | 18 and 26 and 28 |
| 32. | 29 or 30 or 31 |
